# Acalabrutinib (ACP-196): a selective second-generation BTK inhibitor

**DOI:** 10.1186/s13045-016-0250-9

**Published:** 2016-03-09

**Authors:** Jingjing Wu, Mingzhi Zhang, Delong Liu

**Affiliations:** Department of Oncology, The first Affiliated Hospital of Zhengzhou University, Zhengzhou, 450052 China

## Abstract

More and more targeted agents become available for B cell malignancies with increasing precision and potency. The first-in-class Bruton’s tyrosine kinase (BTK) inhibitor, ibrutinib, has been in clinical use for the treatment of chronic lymphocytic leukemia, mantle cell lymphoma, and Waldenstrom’s macroglobulinemia. More selective BTK inhibitors (ACP-196, ONO/GS-4059, BGB-3111, CC-292) are being explored. Acalabrutinib (ACP-196) is a novel irreversible second-generation BTK inhibitor that was shown to be more potent and selective than ibrutinib. This review summarized the preclinical research and clinical data of acalabrutinib.

## Background

New CD20 monoclonal antibodies and bispecific antibodies are providing more treatment options with increasing precision and potency for hematological malignancies [1–7]. Even though chronic lymphocytic leukemia (CLL) remains incurable at this time, more and more treatment options are bringing clinical benefits to patients with longer duration of response and less toxicities commonly seen with conventional chemotherapeutic agents [1, 8–11]. Bruton’s tyrosine kinase (BTK) is an essential kinase in the B cell receptor (BCR) signaling pathway and a driving force for CLL and other B cell malignancies [12–14]. The first-in-class BTK inhibitor, ibrutinib, has been in clinical use for the treatment of chronic lymphocytic leukemia (CLL), mantle cell lymphoma, and Waldenstrom’s macroglobulinemia [11, 13, 15–17]. However, ibrutinib has untoward effects, such as bleeding, rash, and atrial fibrillation, which could be partly due to the bystander effects on targets other than BTK [10, 13, 15, 17, 18]. Therefore, more selective BTK inhibitors (ACP-196, ONO/GS-4059, BGB-3111, CC-292) are being explored [19–23]. Acalabrutinib, also known as ACP-196, is a novel irreversible second-generation BTK inhibitor that was rationally designed to be more potent and selective than ibrutinib [19, 24–28]. This review summarized the preclinical research and clinical data of acalabrutinib.

### Mechanism of action and properties of acalabrutinib

Acalabrutinib binds covalently to Cys481 with improved selectivity and in vivo target coverage compared to ibrutinib and CC-292 in CLL patients [19, 20, 26]. In the in vitro signaling assay on primary human CLL cells, acalabrutinib inhibited tyrosine phosphorylation of downstream targets of ERK, IKB, and AKT [24]. Acalabrutinib demonstrated higher selectivity for BTK with IC_50_ determinations on nine kinases with a cysteine residue in the same position as BTK [19]. Importantly, unlike ibrutinib, acalabrutinib did not inhibit EGFR, ITK, or TEC [19, 24]. In the in vitro assays reported in the supplemental data, it was clearly demonstrated that, unlike ibrutinib, acalabrutinib had no effect on EGFR phosphorylation on tyrosine residues y1068 and y1173. At 1000 nM, ibrutinib completely suppressed Tec activity, though 1000 nM acalabrutinib had minimal activity on Tec [24]. Compared with ibrutinib, acalabrutinib has much higher IC_50_ (>1000 nM) or virtually no inhibition on kinase activities of ITK, EGFR, ERBB2, ERBB4, JAK3, BLK, FGR, FYN, HCK, LCK, LYN, SRC, and YES1 [24].

The differential effects of acalabrutinib on primary CLL cells, T cells, NK cells, and epithelial cells were studied by signaling and functional assays. Acalabrutinib inhibited purified BTK with an IC_50_ of 3 nM and EC_50_ of 8 nM in a human whole-blood CD69 B cell activation assay [19]. Acalabrutinib was shown to have improved target specificity over ibrutinib with 323-, 94-, 19-, and 9-fold selectivity over the other TEC kinase family members (ITK, TXK, BMX, and TEC, respectively) and no activity against EGFR.

The effects of ibrutinib and acalabrutinib on platelets were also compared in an in vivo VWF^HA1^ mouse thrombosis model. The platelets from patients treated with ibrutinib 420 mg once per day or acalabrutinib 100 mg twice per day were assessed for thrombus formation at injured arterioles of the mice. The thrombus sizes from acalabrutinib-treated platelets were comparable to those of healthy controls, whereas thrombus formation was clearly inhibited in ibrutinib-treated platelets. These data suggest that acalabrutinib, unlike ibrutinib, is more selective for inhibiting BTK and has virtually no inhibition of platelet activity [24].

There data clearly suggest that acalabrutinib is a more selective and potent second-generation BTK inhibitor.

### Acalabrutinib (ACP-196) in preclinical research

Acalabrutinib was evaluated in several animal models of B cell non-Hodgkin lymphoma (NHL). These studies provided preclinical in vivo data necessary to move acalabrutinib into human trials. In a study of canine model of B cell NHL, 12 dogs with B cell NHL were orally administered acalabrutinib at escalating dosages of 2.5 mg/kg every 24 h (6 dogs), 5 mg/kg every 24 h (5 dogs), or 10 mg/kg every 12 h (1 dog). As a result, 3 dogs achieved a partial remission (PR), 3 dogs had stable disease (SD), whereas the remaining 6 dogs had progression of disease (PD). This study therefore showed that acalabrutinib has single agent biologic activity in a spontaneous large animal model of NHL [25].

The in vivo effects of acalabrutinib against CLL cells were demonstrated in the NSG mouse model with xenografts of human CLL [28]. Acalabrutinib significantly inhibited proliferation of human CLL cells in the spleens of NSG mice at all dose levels, as measured for the expression of Ki67 (*P* = 0.002). Tumor burden decreased with the treatment of acalabrutinib in a dose-dependent manner. Acalabrutinib inhibited BCR signaling by reduced phosphorylation of PLCγ2. Acalabrutinib transiently increased CLL cell counts in the peripheral blood. Therefore, the novel BTK inhibitor acalabrutinib shows in vivo efficacy against human CLL cells xenografted to the NSG mouse model.

Two murine models were used in another in vivo study [29, 30]. In the TCL1 adoptive transfer model, acalabrutinib inhibited BCR signaling by decreased autophosphorylation of BTK and reduction in surface expression of the BCR activation markers CD86 and CD69. Most interestingly, acalabrutinib treatment increased survival significantly over mice receiving vehicle (median 81 vs 59 days, *P* = 0.02). The second murine model was the NSG xenograft model. Acalabrutinib treatment significantly decreased the phosphorylation of PLCγ2 and ERK (*P* = 0.02), reduced tumor cell proliferation (*P* = 0.02), and tumor burden (*P* = 0.04). Acalabrutinib was shown to be a potent inhibitor of BTK in both murine models of human CLL [29].

### Acalabrutinib in clinical development

To further assess the safety, efficacy, pharmacokinetics, and pharmacodynamics of acalabrutinib in human CLL, a phase 1/2, multicenter, open-label, and dose-escalation clinical trial has been ongoing (NCT02029443). In the latest report, 61 patients with relapsed CLL were enrolled [24]. These patients had received a median of three prior therapies for CLL. Among them, 31 % were positive for 17p13.1 deletion and 75 % had IGvH unmutated. In the phase 1 portion of this study, patients were treated with acalabrutinib at an increasing dose of 100 to 400 mg once daily. In the phase 2 expansion portion, 100 mg twice daily was given. After a median follow-up of 14.3 months (range 0.5–20), the overall response rate (ORR) was 95 %, with 85 % PR, 10 % PR with lymphocytosis. The remaining 5 % of patients were reported to have SD. The ORR was 100 % in those patients with chromosome 17p13.1 deletion. Headache, diarrhea, and weight gain were the most common adverse events. There were no dose-limiting toxicities observed in the phase I portion of the trial. There was no Richter’s transformation and no cases of atrial fibrillation have been reported to date [24]. The preclinical data from this report confirmed that acalabrutinib is a highly selective BTK inhibitor. It does not inhibit TEC kinase and platelet aggregation which would be a preferred advantage over ibrutinib and would have reduced bleeding risk. Acalabrutinib does not inhibit EGFR, thus could reduce the adverse events on skin rash and severe diarrhea. Transient headaches were reported to be a common adverse event from acalabrutinib and appeared to be more frequently seen than in those patients on ibrutinib from historical data. More patients and longer follow-up from this study are needed to ascertain these potential advantages and disadvantages. In addition, direct comparison of acalabrutinib with ibrutinib will be the only way to confirm the advantages and disadvantages of these agents.

At this time, a phase 3 study (NCT02477696) has commenced in which acalabrutinib is being compared with ibrutinib in high-risk patients with relapsed CLL. Studies in treatment-naïve CLL are also being done (Table 1).

**Table 1:** Acalabrutinib (ACP-196) for hematological malignancies

Agents	Diseases	Phase	NCT
ACP-196 Dexamethasone	MM	Phase 1	NCT02211014
ACP-196	ABC DLBCL	Phase 1	NCT02112526
ACP-196 Rituximab (IV)	FL	Phase 1	NCT02180711
ACP-196 Obinutuzumab	CLL, SLL, PLL	Phase 1	NCT02296918
ACP-196 ACP-319	CLL	Phase 1	NCT02157324
ACP-196 ACP-319	NHL, MM, B-All	Phase 1/2	NCT02328014
ACP-196	WM	Phase 1/2	NCT02180724
ACP-196	CLL, SLL, RS, PLL	Phase 1/2	NCT02029443
ACP-196 Pembrolizumab	NHL, MM, HL, CLL, RS, WM, Myelofibrosis	Phase 1/2	NCT02362035
ACP-196	CLL, SLL	Phase 2	NCT02337829
ACP-196	MCL	Phase 2	NCT02213926
ACP-196 Obinutuzumab Chlorambucil	CLL	Phase 3	NCT02475681
ACP-196 Ibrutinib	CLL	Phase 3	NCT02477696

*NHL* non-Hodgkin lymphoma, *HL* Hodgkin lymphoma, *MM* multiple myeloma, *MCL* mantle cell lymphoma, *FL* follicular lymphoma, *ABC DLBCL* activated B cell diffuse large B cell lymphoma, *CLL* chronic lymphocytic leukemia, *SLL* small lymphocytic lymphoma, *WM* Waldenstrom’s macroglobulinemia, *RS* Richter’s syndrome, *PLL* prolymphocytic leukemia

## Conclusion and future directions

Acalabrutinib is a more selective irreversible second-generation BTK inhibitor. It has improved target specificity and enhanced potency for BTK due to reduced off-target activity on EGFR, TEC, etc., which may lead to less untoward effects and toxicities. More patients and longer follow-up from the ongoing phase I/II clinical study are needed to ascertain these potential advantages. Multiple trials on other hematological malignancies and solid tumors are underway (Tables 1 and 2). Combination of acalabrutinib and other agents (new CD19 and CD20 antibodies, BCL-2 inhibitors, PI3 kinase inhibitors, ALK inhibitors, etc.) for B cell malignancies will likely further increase response rate and duration of response [1, 8, 31–37] (Table 1). It will also be interesting to see whether resistance mechanisms will be different from those for ibrutinib. Finally, additional selective BTK inhibitors, i.e., ONO/GS-4059 and BGB-3111, are under active clinical development [21–23]. More options of treatment may become available for B cell malignancies.

**Table 2:** Acalabrutinib (ACP-196) for solid tumors

Agents	Diseases	Phase	NCT
ACP-196	Glioblastoma multiforme	Phase 1/2	NCT02586857
ACP-196 Pembrolizumab	Urothelial carcinoma	Phase 2	NCT02351739
ACP-196 Pembrolizumab	Non-small-cell lung cancer	Phase 2	NCT02448303
ACP-196 Pembrolizumab	Head and neck Squamous cell carcinoma	Phase 2	NCT02454179
ACP-196 Pembrolizumab	Ovarian cancer	Phase 2	NCT02537444
ACP-196 Pembrolizumab	Pancreatic cancer	Phase 2	NCT02362048
ACP-196 Nab-paclitaxel Gemcitabine	Pancreatic cancer	Phase 2	NCT02570711
